# Applications of Optically Controlled Gold Nanostructures in Biomedical Engineering

**DOI:** 10.3389/fbioe.2020.602021

**Published:** 2021-01-20

**Authors:** Pisrut Phummirat, Nicholas Mann, Daryl Preece

**Affiliations:** ^1^Department of Biomedical Engineering, University of California, Irvine, Irvine, CA, United States; ^2^Beckman Laser Institute, University of California, Irvine, Irvine, CA, United States

**Keywords:** optical forces, nanomanipulation, cell biology, GNPs, nanoscience, biomedical engineering, gold nanoparticles, optical tweezers

## Abstract

Since their inception, optical tweezers have proven to be a useful tool for improving human understanding of the microscopic world with wide-ranging applications across science. In recent years, they have found many particularly appealing applications in the field of biomedical engineering which harnesses the knowledge and skills in engineering to tackle problems in biology and medicine. Notably, metallic nanostructures like gold nanoparticles have proven to be an excellent tool for OT-based micromanipulation due to their large polarizability and relatively low cytotoxicity. In this article, we review the progress made in the application of optically trapped gold nanomaterials to problems in bioengineering. After an introduction to the basic methods of optical trapping, we give an overview of potential applications to bioengineering specifically: nano/biomaterials, microfluidics, drug delivery, biosensing, biophotonics and imaging, and mechanobiology/single-molecule biophysics. We highlight the recent research progress, discuss challenges, and provide possible future directions in this field.

## 1. Introduction

Optical tweezers have seen a multitude of technological developments over the past decades that have improved the functionality and flexibility of the technology first created by Ashkin et al. (1986). These systems have been instrumental in opening up new areas of biomedical research and allowing researchers new ways to manipulate and investigate a variety of interesting biological phenomena.

In conventional optical tweezers systems a high NA microscope objective is used to create a highly focused laser beam that possesses the strong field gradients necessary to form a stable trap. The forces experienced by the trapped object consists of the light scattering and gradient forces caused by light-matter interactions. These resulting optical forces typically ranging between 0.1 and 100 pN, enough to move small (1–10 μm) polystyrene or silica beads (Ashkin et al., 1986).

In recent years many scientists have utilized metallic nanostructures, such as gold nanoparticles (GNPs), for optical micromanipulation due to their large polarizability and relatively low cytotoxicity (Jauffred et al., 2019). Though conventionally much smaller than silica or PS beads, metallic nanostructures can also be trapped using the optical gradient force (Svoboda and Block, 1994). For 3D optically trapped GNPs with diameters between 18 and 254 nm, the trapping strength increases with radius of the beads (Hansen et al., 2005). Shape also matters in that for non-spherical GNPs, such as gold nanorods (Pelton et al., 2006) or nanoaggregates (Messina et al., 2011), plasmon resonances in the visible/NIR region can play a crucial role in OT by enhancing the gradient force if the trapping laser is tuned to the long-wavelength side.

However, strong field gradients are necessary to counteract the effects of smaller optical cross sections and increased diffusion. Nanoparticles may also be subject to a variety of other anomalous forces created by thermal, electrostatic and chemical interactions. This has motivated several new methodologies to produce stronger optical confinement by exploiting novel trapping mechanisms, such as near-field forces, nanoapertures (Gordon, 2019), plasmonic fields (Ghosh and Ghosh, 2019), hydrodynamic flows (Būtaitė et al., 2019), and others (Hansen et al., 2005; Hajizadeh and Reihani, 2010).

In this article we will review the progress made in applying optically controlled gold nanomaterials for applications in biomedical engineering. We will not deal extensively with expositions of physical phenomena since several papers exist which deal with the physics of optical trapping in detail. Rather we will concentrate on surveying the newest applications of the technology to real world problems, as the authors could find few papers or reviews on this subject area. We anticipate that this paper will help scientists and engineers direct new research efforts into emerging biomedical problems and also yield insights into to how previous studies have applied the technology.

## 2. Applications in Biomedical Engineering

### 2.1. Optical Control of Nanoparticles

**Figure 1:**
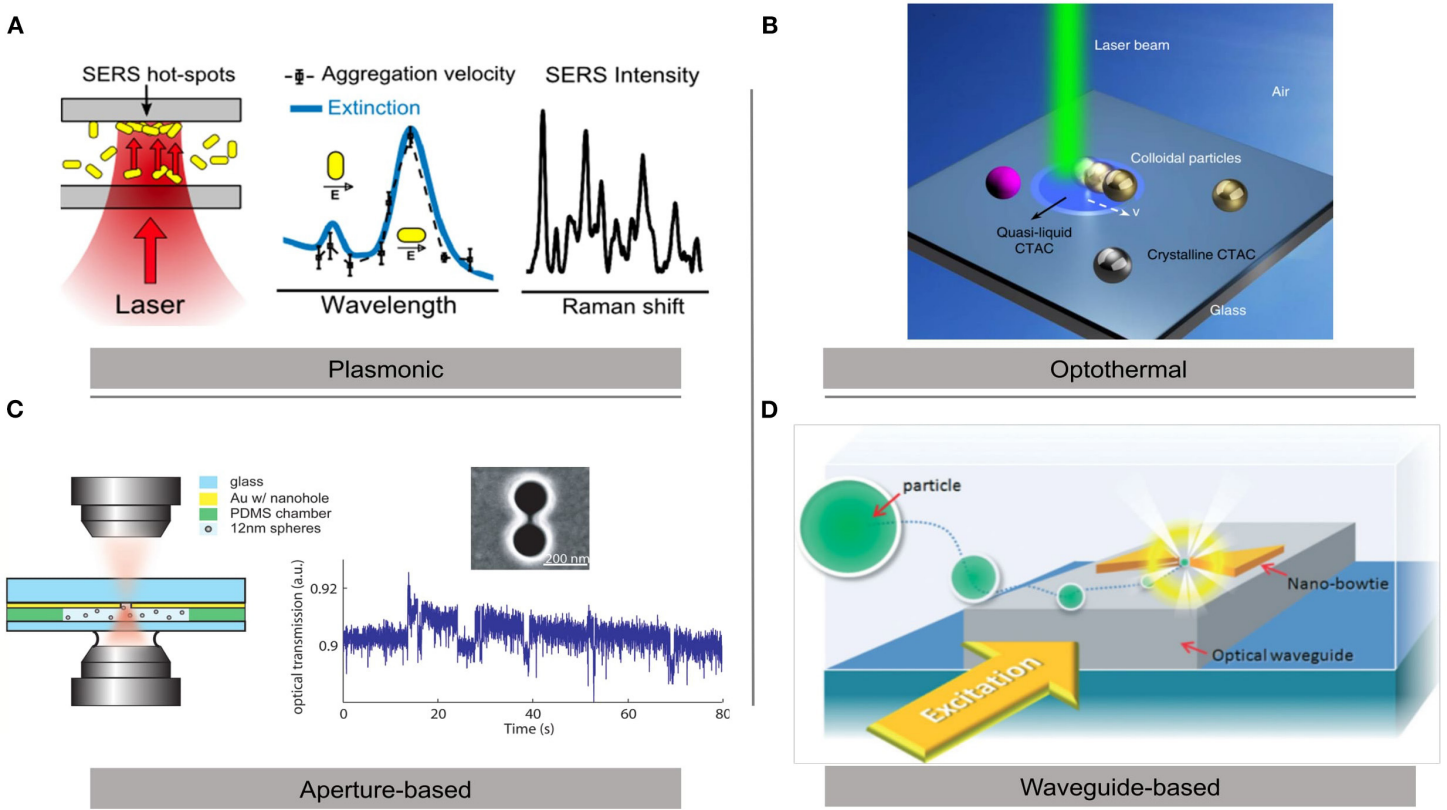
Different schemes for optical trapping of gold nanoparticles. **(A)** Plasmonic optical tweezers for microfluidics applications (Bernatová et al., 2019). **(B)** Optothermal optical tweezers for nanomanipulation applications (Li et al., 2019a). **(C)** Double nanohole aperture optical tweezers for single-molecule studies (Pang and Gordon, 2011). **(D)** Waveguide-coupled gold bowtie plasmonic tweezers for lab-on-chip developments (Lin et al., 2014). Copyright 2019 ACS, 2019 NR, 2011 ACS, and 2014 RSC.

Several methodologies have been recently developed to optically control nanoparticles (Figure 1). One common approach is to utilize deposited metal structures to enhance electric field gradients. Classically “Bowtie” plasmonic structures have been used in this regard (Lin et al., 2014) but other shapes have also proven useful. Nanoapertures have been used to enhance field gradients while maintaining particle specificity (Pang and Gordon, 2011; Gordon, 2019). Other shapes, such as nanodisks utilizing plasmonic resonances have also been used to trap nanoparticles (Jiang et al., 2019). Kotsifaki et al. (2020) used Fano resonance-assisted plasmonic optical tweezers for single nanoparticle trapping in an array of asymmetrical split nanoapertures. Such approach may limit control of particles to geometrically defined areas. Hoshina et al. (2018) proposed a scheme to trap the NPs into a particular hotspot of the metallic nanostructure array utilizing structured light to control particle position. Gao et al. (2019) demonstrated that dynamic holographic optical tweezers are capable of manipulating single microparticles in a gold coated microfluidic sample cell with the precision and stability required for coherent X-ray diffraction imaging. Another novel approach is to use plasmonic nanodisks over dielectric microrods. These hybrid structures can be controlled by conventional OT and at the same time give strongly confined optical near-fields in their vicinity (Ghosh and Ghosh, 2019).

In order to create strong optical forces, optical standing waves can also be used to trap GNPs directly. Schnoering et al. (2019) proposed a novel optical force microscopy based on a standing-wave optical trap. Standing-wave Raman tweezers allow for characterization of individual NPs, including biological particles, which is otherwise difficult using single-beam tweezers (Wu et al., 2017).

### 2.2. Optical Printing and Assembly of Bio/nanomaterials

Optical forces are a powerful tool for micro-/nano-assembly, as they offer non-contact, bio-friendly techniques for maneuvering a variety of objects of different sizes and material properties in three dimensions. Assembling structures, however, can be challenging. Metal nanomaterials offer a viable solution for large scale assemblies due to their remarkably strong optical binding forces which promote self assembly of nanostructures. Nanostructures can then be used for sensors, nanophotonic structures, or by themselves as a printable substrate.

Patterning nanostructures with optical fields presents a relatively robust way of precision printing (Guffey and Scherer, 2010; Urban et al., 2010; Gargiulo et al., 2017). These prints can achieve remarkable precision but require a sophisticated understanding of optical, chemical and electrostatic forces. Large scale prints have also been difficult to achieve. Ions and nanomaterials can be directly assembled in an optically controlled assembly chip (Liu et al., 2016) or directed by exploiting phase gradients (Rodrigo and Alieva, 2016). Ota et al. first reported lipid bilayer-integrated optoelectronic tweezers for simultaneous manipulation of many GNPs in desired patterns. Through the NPs tethered on the supported lipid bilayer, this method could facilitate mechanotransduction studies and molecular sensing at the cellular interface (Ota et al., 2013).

Li et al. (2019a) constructed colloidal gold particles into functional nano-structures on solid substrates via an all-optical technique called optothermally-gated photon nudging (OPN). Recently, *in-situ* construction of permanent mesoscale structures from optically bound nanoparticles was reported (Chen et al., 2020). Plasmonic NPs are trapped by OT and self-organized into a variety of optically bound structures, which are then immobilized by UV activated hydrogels. Perhaps of greater interest to bioengineers is the assembly of more complicated heterogeneous structures. Kang et al. (2015) used gold nano-islands to optically trap and assemble particles and live cells into highly organized patterns.

Optically manipulated biophotonic structures also find applications in sensing and control applications for soft materials, such as proteins and DNA. Shoji and Tsuboi describe the developments of plasmonic optical tweezers for soft nanomaterials. The combination of resonant optical trapping with nano patterned plasmonic surfaces permits small molecule manipulation, such as heme proteins (Shoji and Tsuboi, 2014) the photothermal effect of AuNPs can be harnessed to drive phase separation of polymer solution along with assembling and swarming of AuNPs simultaneously (Aibara et al., 2020).

Optical tweezers are a powerful tool for printing and assembly of GNPs. While it may have lower yields compared with other conventional means, such as chemi/physisorption or photolithography, more precise control over the position and configuration of NPs can be advantageous (Table 1). To overcome the electrostatic repulsion to the substrate, NPs need to have the right size to interact strongly with the beam focus.

**Table 1:** Comparison between different optical tweezers methodologies.

**Trapping mechanism**	**Heating**	**High spatial resolution**	**Scalability**	**Selectivity**	**Sorting**	**Diffusion limited**	**References**
Conventional	√		√	√	√		Guffey and Scherer, 2010; Urban et al., 2010; Arita et al., 2013; Aekbote et al., 2014; Wagner et al., 2016; Gargiulo et al., 2017; Gao et al., 2019; Misra et al., 2019; Chen et al., 2020; Vizsnyiczai et al., 2020
Plasmonic	√	√			√	√	Shoji and Tsuboi, 2014; Hoshina et al., 2018; Bernatová et al., 2019; Ghosh and Ghosh, 2019; Aibara et al., 2020; Kotsifaki et al., 2020; Rickard et al., 2020
Optothermal		√	√		√		Kang et al., 2015; Villangca et al., 2016; Šípová et al., 2018; Li et al., 2019a; Kotnala et al., 2020
Optoelectronic		√	√		√		Maruyama et al., 2011; Ota et al., 2013
Waveguide-based	√	√	√		√	√	Lin et al., 2014
Aperture-based		√		√		√	Pang and Gordon, 2011; Kotnala and Gordon, 2014; Gordon, 2019; Jiang et al., 2019; Burkhartsmeyer et al., 2020

### 2.3. Applications in Microfluidics and Particle Sorting

It is perhaps unsurprising that manipulation of GNPs has found applications in microfluidics and cell sorting. Microfluidics have been a workhorse in many biotechnology applications for decades. However, the popularity of nanoparticle based sensing applications has created a need for integration of optical technologies with lab on a chip type systems. A recent study in Nature Biomedical Engineering proposed a SERS integrated optofluidic device for fast detection of traumatic brain injury (TBI) biomarkers in finger-prick blood plasma at picomolar concentrations (Rickard et al., 2020). Bernatova et al. used optical forces to obtain SERS-active hotspots inside a microfluidic chip. Gold nanorod aggregates were created in the presence of protein, and metal-nanoparticle-protein complexes were printed in microfluidic chips (Bernatová et al., 2019). This SERS biosensor can serve as the detection part in microfluidic micro-assays or lab-on-chip devices. Another study combined OT and DEP for finding the concentration of viruses on a microfluidic chip (Maruyama et al., 2011).

Optically driven systems provide several advantages when integrated into microfluidic systems. One application is cell sorting. Cell sorters require high purity and recovery rates. Optical tweezers have proven useful for small sample sizes (Wang et al., 2011). Arbitrary cells, including bacteria, yeast, and mitochondria, can be sorted inside a microchannels at different displacement speeds or frequencies using fast steerable optical traps (Landenberger et al., 2012). Nanoparticle sorting is far more challenging due to particle movement caused by Brownian diffusion of particles, low throughputs, and challenges detecting particles (Salafi et al., 2017).

Tsuji et al. (2019) demonstrate that using optical forces, nanoparticle flow can be controlled in all-quartz glass nanoslit channels. Yin et al. (2020) have also proposed a plasmonic nanoparticle router, which consists of a series of gold nanostrips on top of a continuous gold thin film, to transport trapped nanoparticles along designated routes in a microfluidic channel with a continuous flow under the incident unfocused light.

A more promising way of controlling nanoparticles is to use fluid forces and generating flows indirectly. Instead of using intense lasers, Būtaitė et al. (2019) demonstrate a trapping platform which harnesses hydrodynamic forces generated from light driven micro-rotors to manipulate aqueous mesoscale particles with nanoscale-precision. By combining concepts from both optical and hydrodynamic approaches, a fully re-configurable system capable of inducing highly localized flow fields targeted only at specific particles, many of which cannot be directly optically tweezed, is realized. An integrated optofluidic micro-pump, driven by plasmon-assisted optical manipulation with H-shaped gold apertures, was used along with a single polarization rotating beam to create the circulation of hot spots, which can trap beads and make them rotate (Jiang et al., 2019).

Optically generated microbubbles can also be used to manipulate nanoparticles. This was first demonstrated by Berry et al. (2000) and Yusupov et al. (2014). Recently Kotnala et al. (2020) has exploited this idea to create nanoaperture-based plasmonic sensors using gold nano-islands. Convective flow decreases trapping times by 1–2 orders of magnitude enhancing sensitivity and throughput of nanoaperture-based plasmonic sensors.

Single particle manipulation is of great interest to many researchers as various schemes have been proposed in the literature. Although optical based microfluidic systems can be bulky and require high optical powers, they can be made more compact and user-friendly to attract more non-experts. Also, a more synergistic combination of optical forces and microfluidics can lead to higher throughput.

### 2.4. Targeted Drug Delivery and Nanorobotics

Despite few reports associated with toxic effects, colloidal gold nanoparticles, chemically inert, and biocompatible, have attracted widespread interest for drug delivery in humans (Pan et al., 2007; Sung et al., 2011). Unlike biopolymeric nanomaterials, metal nanoparticles, including gold, exhibit properties like surface plasmon resonance; liposomes, dendrimers, and micelles do not. GNPs can be loaded within these molecules (Kong et al., 2017) or therapeutic drugs are attached to GNPs (Paasonen et al., 2007) and delivered to target cells or tissues in a controlled fashion. Perhaps the most publicized use of GNPs in drug delivery research is cancer therapy/diagnosis. In fact, Aurimune (CYT-6091), PEGylated GNPs based nanomedicines developed by Astra Zeneca in partnership with CytImmune, are currently under clinical trials (CytImmune, 2020).

Early work in this area of research was performed when single 100 nm gold NPs were optically tweezed and injected into a specified region of a mammalian cell's interior for the first time (McDougall et al., 2009). More recent work on optical injection into living cells utilize 80 nm gold beads (Urban et al., 2011; Li et al., 2015; Maier et al., 2018). One attractive approach toward this goal is plasmonic nanoparticle-based drug delivery (Sharifi et al., 2019). With a myriad of existing ways to conjugate molecules to metal surfaces, particularly gold, strategies for controlled release of drugs and therapeutic agents from nanostructures upon laser irradiation abound.

A novel dynamic optical nanomanipulation scheme was devised by using plasmonic nanodisks over dielectric microrods. These hybrid structures can be controlled by conventional OT and at the same time give strongly confined optical near-fields in their vicinity as in plasmonic tweezers. The colloidal tweezers can be used to transport cargo as small as 40 nm in ionic solutions using lower optical power than conventional Gaussian beam tweezers (Ghosh and Ghosh, 2019). A nanopipette based on optothermophoretic fiber tweezers (OTFT) has been demonstrated to successfully deliver 200 nm gold nanoparticles to a single large unilamellar lipid vesicle (Kotnala and Zheng, 2019).

Particulate materials that selectively localize to a specific cellular subunit are of interest for drug delivery applications. Recently, the novel idea of preferential partitioning of hierarchically assembled particles with surface-bound amphiphilic gold nanoparticles to cell membranes of living cells using OT was realized (Misra et al., 2019). Villangca et al. (2016) used light to generate and control secondary hydrodynamic effects by heating an embedded thin gold layer within the trapped microstructure, which resembles a racing car in shape. Thermal convection currents inside the microtool draw fluid along with potential cargo in or out of its body.

A single gold nanoparticle was optically trapped and subsequently subject to laser-induced breakdown and gentle nanocavitation upon irradiation by a ns-laser pulse. In so doing, transfection of plasmid-DNA into individual cells was performed with 75% efficiency (Arita et al., 2013). 10–250 nm gold beads can be heated upon optical trapping in the contact zone, causing a total fusion of two adjacent vesicles of interest, which is highly useful for single-cell targeted drug delivery and hybrid cell creation (Rørvig-Lund et al., 2015).

Different drug delivery systems have pros and cons. While bioavailability and unintended side effects can limit the use of GNPs for clinical applications, optical based targeted DDSs are unique in that light can be tuned to direct the movement of carriers and release the loaded drugs.

### 2.5. Biosensing and Bioimaging

Biosensors are defined as analytical devices incorporating a biological material, a biologically derived material or a biomimetic intimately associated with or integrated within a physicochemical transducer or transducing microsystem (Turner et al., 1989). AuNPs are widely used in the field of bioassay. Non-optical bioassays include electrochemical (Raj and Jena, 2005; Andreescu and Luck, 2008) and piezoelectric biosensors (Liu et al., 2004).

Optical biosensors are one of the most common types of biosensor. They provide advantages over other analytical approaches due to their direct, real-time and label-free detection of many biological and chemical substances (Damborský et al., 2016). However, incorporation of optical manipulation into sensors is rare. Gordon (2019) have used gold nanoaperture optical tweezers to perform manipulation, sensing and spectroscopy of biological nanoparticles below 50 nm in size. Another interesting application of trapped nanoparticles is to measure photothermal DNA release from rationally controlled gold nanomotors. By using rotational dynamics analysis, Šípová et al. (2018) resolve differences in the thickness of absorbed ultrathin molecular layers, including different DNA conformations, with nanometer resolution.

The Raman spectroscopy in combination with tweezers based technology has provided a way to make otherwise difficult measurements easier. For example, Barkur et al. (2020) measured hemoglobin deoxygenation in live RBCs. This technology can also be used with SERS particles (Jiang et al., 2019) which can be manipulated to provide measurements at different locations. Another option is to use fiber based optical systems which can be used as probes to sense specific locations. Chen et al. (2018) used U-Shaped fiber probe coated with GNPs and glucose oxidase to detect blood glucose. Li et al. (2019b) built a fiber based biomagnifier which could trap and magnify nano-objects with subdiffraction-limited resolution.

GNPs have also contributed to the field of bio-optical imaging in large part due to their unique plasmonic properties. Based on their properties, such as absorption, scattering, fluorescence, or Raman scattering, optical imaging can be enhanced. Issues associated with resolution, sensitivity, penetration depth can be improved upon by using GNPs (Wu et al., 2019). Optically controlled gold coated nanotools were employed to achieve localized fluorescence enhancement (Aekbote et al., 2014). Similarly, the fluorescence of optically trapped NPs was key to realize a sub-200 nm resolution with a visible light (Wagner et al., 2016). Later, Vizsnyiczai et al. presented a multiview microscopy of single cells based on holographic optical tweezing of microstructures made with two-photon polymerization. The presented tool allows for precise manipulation of the cells in 6 degrees of freedom and solves the axial resolution problem that comes with fluorescence imaging (Vizsnyiczai et al., 2020).

Although non-optical based biosensors have simpler detection schemes, those of GNP based optical biosensors are faster and more clinically relevant. As for bioimaging, contrast agents, such as fluorescent probes have made visualizing specific biological processes possible and become mainstream in modern biomedical research. Yet optically controlled GNPs based techniques may offer enhanced bioimaging.

### 2.6. Mechanobiology and Single-Molecule Biophysics

OT have been used to manipulate matter in a non-invasive way for studies like measuring macromolecular interactions in colloidal systems or studying the forces exerted by molecular motors. Mechanical properties, such as the membrane elasticity and viscoelasticity of DNA are also evaluated. In fact, optical trapping is currently one of the highly preferred tools for manipulating microscopic objects in single-molecule biophysics. A wide array of biochemical processes have been explored using OT, such as molecular motors (Spudich et al., 2011), DNA-protein binding (Heller et al., 2014), protein folding dynamics (Cecconi et al., 2005), ribosome motion during translation (Qu et al., 2011), and RNA polymerase motion during transcription (Fazal et al., 2015).

Nanoaperture trapping has emerged as a useful, label-free tool for single-molecule biophysics studies (Juan et al., 2009; Pang and Gordon, 2011; Chen et al., 2012; Al Balushi et al., 2013; Berthelot et al., 2014). Using double-nanohole optical tweezers, Kotnala and Gordon studied single protein-DNA interactions and demonstrated, they believe for the first time, the direct role of tumor suppressor protein p53 in suppressing DNA unzipping (Kotnala and Gordon, 2014). Burkhartsmeyer et al. (2020) trapped a single viral particle, the bacteriophage PhiX174, as small as 25 nm in diameter via double-nanohole apertures in a gold film to measure its vibrational frequencies, as opposed to large ensembles of particles as in prior optomechanical techniques.

At around the same time, Ma et al. (2020) tethered a nanoparticle with a single DNA molecule to a gold surface for studying the entropic and damping forces. A recent study conducted by Kotsifaki et al. takes advantage of Fano resonance-assisted plasmonic optical tweezers (FAPOT) for single nanoparticle trapping in an array of asymmetrical split-ring (ASR) metamaterials on a gold film. Significantly enhanced trap stiffness results from the strong interaction between the trapped nanoparticle and the surface plasmon metamaterial resonance offering new alternatives for studying transition paths of single biomolecules, such as the folding of protein or nucleic acids (Kotsifaki et al., 2020).

Compared with other commonly used single-molecule manipulation methods (AFM and magnetic tweezers), OT are arguably the most versatile technique that can exert forces in excess of 100 pN with sub-nm accuracy and sub-ms time resolution. These characteristics make OT particular attractive for subcellular force and motion studies. While radiation pressure is reduced with decreasing trapped object size and thermal fluctuation can become non-negligible, novel techniques are emerging to address them.

## 3. Conclusion

As we have shown gold nanostructures have found many applications in bioengineering from the manipulation of single molecules to novel sensing applications. What is more, optical forces have proven useful for biomedical applications due to the largely the passive nature of light on many biological systems. GNPs in particular have proven to be useful targets for optical manipulation. The unique resonant properties of such particles have found many applications in biotechnology.

Understanding the evolution of optical fields in nanoscale metallic structures is still complicated, notably when particles are arranged in complex or composite shapes or structures. Substantial theoretical work has been devoted to this subject but there is still much to do in the future. Despite its challenges, optical manipulation at the nanoscale has found increasing biological applicability. The use of GNPs has only enhanced this. Single molecule studies and the investigation of biochemical interactions at the nanoscale provide fertile ground for future study.

The authors look forward with interest to see how this dynamic field of study develops in the future.

## Author Contributions

PP, NM, and DP wrote the manuscript. DP and PP conceived the idea for the paper. PP and NM researched the paper. All authors contributed to the article and approved the submitted version.

## Conflict of Interest

The authors declare that the research was conducted in the absence of any commercial or financial relationships that could be construed as a potential conflict of interest.
